# Characterization of the role of the tumor marker Nup88 in mitosis

**DOI:** 10.1186/1476-4598-9-119

**Published:** 2010-05-24

**Authors:** Chieko Hashizume, Hiroshi Nakano, Kimihisa Yoshida, Richard W Wong

**Affiliations:** 1 Frontier Science Organization and Cancer Research Institute, Kanazawa University, Kakuma-machi, Kanazawa, Ishikawa, 920-1192 Japan; 2 Laboratory of Cell Biology, Howard Hughes Medical Institute, The Rockefeller University, 1230 York Avenue, New York, NY 10065, USA

## Abstract

Nuclear pore complexes are massive multiprotein channels responsible for traffic between the nucleus and cytoplasm, and are composed of approximately 30 proteins, termed nucleoporins (Nup). Our recent studies indicated that the nucleoporins Rae1 and Tpr play critical roles in maintaining the spindle bipolarity during cell division. In the present study, we found that another nucleoporin, Nup88, was localized on the spindles together with Nup214 during mitosis. Nup88 expression is linked to the progression of carcinogenesis, Nup88 has been proposed as a tumor marker. Overexpression of Nup88 enhanced multinucleated cell formation. RNAi-mediated knockdown of Nup88 disrupted Nup214 expression and localization and caused multipolar spindle phenotypes. Our data indicate that proper expression of Nup88 is critical for preventing aneuploidy formation and tumorigenesis.

## Findings

The nuclear envelope forms a physical selective barrier between the nucleus and cytoplasm, and controls protein, RNA and ribonucleoprotein transportations in eukaryotic cells. Nucleocytoplasmic transport is exclusively mediated by nuclear pore complexes (NPCs), which are large proteinaceous channels that span the nuclear envelope. Vertebrate NPCs are composed of about 30 proteins, termed nucleoporins (Nups), which are present in multiple copies. Despite differences in the overall sizes in different species, the basic architecture of NPCs is well conserved among species. NPCs/Nups localization is very dynamics. In higher eukaryotes, NPCs are disassembled during cell division. We found that nucleoporins (Rae1 and Tpr) play critical roles in maintaining the spindle bipolarity during mitosis [1-4]. On the other hand, during interphase, pore proteins or nucleoporins [5,6] (designated Nup followed by their predicted molecular weight) are modular in that a limited number of structural motifs (coiled-coils, α-solenoids and β-propellers) are used repeatedly to build the symmetrical NPC channels on the nuclear membrane [5]. Approximately one-third of nucleoporins contain a domain of phenylalanine-glycine (FG) motifs interspersed with spacer sequences. These repeat domains are natively unstructured and serve as interaction sites for transport receptors (karyopherins) that escort cargos through the pore. For more information on NPC structure and function, a number of excellent reviews are available [7,8].

In the past few years, several components of NPCs have been revealed to play important roles during mitosis [8-14]. In particular, we demonstrated that a nucleoporin, RNA export factor 1 (Rae1), interacted with NuMA [3] and cohesin subunit SMC1 [1,2] during mitosis, and played crucial roles in proper spindle formation. Interestingly, a recent report showed that during Vesicular stomatitis virus (VSV) infection or in the presence of M protein alone, cells can undergo death during mitosis after inhibiting spindle assembly and nuclear formation, which involves disruption of Rae1 functions [15].

**Figure 1:**
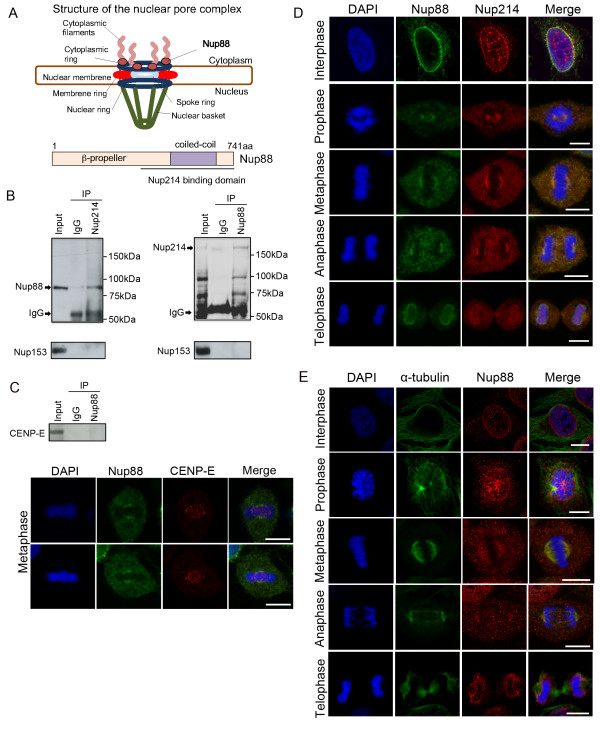
**Nup88 and Nup214 form a complex during mitosis**. (A) Schematic diagrams of the structure of the nuclear pore complex (upper) and the nucleoporin Nup88 domains organization (lower). (B) Immunoprecipitates from mitotic HeLa cell extracts with anti-Nup214, anti-Nup88 antibodies or nonspecific rabbit antibodies (IgG) were analyzed by SDS/PAGE, followed by immunoblotting with an anti-Nup88, anti-Nup214 or anti-Nup153 antibodies respectively. In the lanes marked ''input'', 20 μl of the 500-μl extract that was used per immunoprecipitation was analyzed directly. (C) Immunoprecipitates from mitotic HeLa cell extracts with anti-Nup88 antibodies or nonspecific rabbit antibodies (IgG) were analyzed by SDS/PAGE, followed by immunoblotting with an anti-CENP-E antibody (upper panel). HeLa cells were costained with anti-Nup88 (green) and anti-CENP-E (red) antibodies and analyzed by confocal laser microscopy. Chromatin was visualized using DAPI (blue)(Lower panel). (D) HeLa cells were costained with anti-Nup88 (green) and anti-Nup214 (red) antibodies and analyzed by confocal laser microscopy. Chromatin was visualized using DAPI (blue). (E) HeLa cells were costained with anti-α-tubulin (green) and anti-Nup88 (red) antibodies and analyzed by confocal laser microscopy. Chromatin was visualized using DAPI (blue). Scale bar, 10 μm.

Nup88 is a non-FG nucleoporin found exclusively on the cytoplasmic face of NPCs [16]. Nup88 has no sequence homology to known proteins. Its N-terminal domain is predicted to form a β-propeller and its C-terminus contains sequences that are predicted to form a coiled-coil domain (Figure 1A). Nup88 interacts with the FG repeat nucleoporin CAN/Nup214 [17,18], another nucleoporin and a proto-oncogene implicated in leukemia [16]. Both the FG repeat domain of Nup214 and the N-terminal β-propeller domain of Nup88 bind directly to CRM-1/exportin-1, the receptor for export of most proteins from the nucleus [16,18]. Nup88 also interacts with the other FG nucleoporins Nup358 [19] and Nup98 [16]. In tumors, Nup88 staining is prominent in the cytoplasm, often in granular dots. Staining is especially evident in carcinomas but is also observed in sarcomas, lymphomas and mesotheliomas [20]. Furthermore, its expression levels are highly correlated with the metastasis and mortality rates of colon cancer and the aggressiveness of breast cancer [21,22]. Since Nup88 expression is linked to the progression of carcinogenesis, Nup88 has been proposed as a tumor marker [16]. However, the functional consequences of Nup88 overexpression in cancer remain unknown.

To further clarify the specific role of Nup88 in mitosis, we analyzed the composition of purified Nup88 complexes in mitotic HeLa cells. Nup88 was reported to interact with the FG repeat nucleoporin CAN/Nup214, another nucleoporin and a proto-oncogene implicated in leukemia during interphase [16]. In immunoprecipitation experiments (Additional file 1), an anti-Nup88 antibody immunoprecipitated Nup214 but not Nup153 (Figure 1B). Conversely, an anti-Nup214 antibody immunoprecipitated Nup88 but not Nup153 (Figure 1B). These data suggest that Nup88 and Nup214 interact during mitosis. Consistent with the immunoprecipitation data, we found that Nup88 and Nup214 colocalized in HeLa cells during the cell cycle (Figure 1D). These experiments revealed a stable association of Nup214 and Nup88 during the whole cell cycle. To examine the Nup88 topography with respect to the mitotic apparatus, we used specific antibodies against Nup88, α-tubulin (spindle marker) and CENP-E (kinetochore marker) to examine their localizations at different stages of the cell cycle. Immunofluorescence microscopy (Additional file 1) of HeLa cells during interphase revealed that Nup88 was predominantly distributed on the nuclear envelope, with typical nuclear rim staining (Figure 1D and 1E, upper panel), whereas α-tubulin was mainly localized in the cytoplasm (Figure 1E, upper panel). Moreover, we could not immunoprecipitate CENP-E nor co-localize with this kinetochore marker, our data indicated that Nup88 was not mainly localized on the kinetochores during mitosis (Figure 1C). Although, colocalization of Nup88 and α-tubulin in the interphase cytoplasm was relatively weak (Figure 1E, yellow areas in merged images); we found that at early prophase, Nup88 and microtubules were concentrated at the vertices of the developing spindle poles (Figure 1E). From late prophase through anaphase, Nup88 and α-tubulin were enriched in a crescent-shaped area and intensely stained at the spindle and spindle poles (Figure 1E, metaphase, anaphase). At telophase, Nup88 was detected in the cytoplasm as well as in the newly developed nuclear envelope membrane, whereas α-tubulin was mainly associated with midzone microtubules (Figure 1E). No staining was apparent when primary antibodies were replaced by pre-immune rabbit or mouse IgG (data not shown). We examined endogenous Nup88 in ≈100 interphase cells and ≈100 cells in each stage of mitosis in three separate experiments. Consequently, these results suggest that Nup88 partially colocalizes with α-tubulin in the cytoplasm of interphase cells and spindles during mitosis.

**Figure 2:**
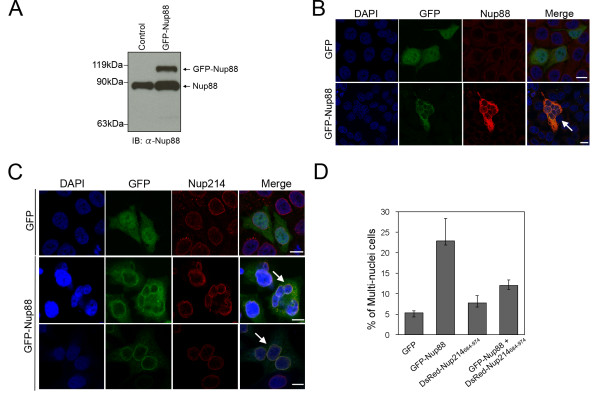
**Overexpression of GFP-Nup88 and Nup214_684-974 _rescues spindle bipolarity**. (A) HeLa cells were transfected with a GFP-Nup88 expression plasmid. After 48 h, the cells were lysed and analyzed by immunoblotting with an anti-Nup88 antibody. (B) Representative images of mitotic HeLa cells transfected with the plasmid overexpressing GFP-Nup88 (full-length) or GFP vector alone. At 48 h after transfection, the cells were fixed, stained with an anti-Nup88 antibody (red) and analyzed by confocal laser microscopy. Chromatin was stained with DAPI (blue). Scale bar, 10 μm. The white arrow indicates a typical multinucleated cell. (C) Representative images of mitotic HeLa cells transfected with the plasmid overexpressing GFP-Nup88 (full-length). The cells were fixed, stained with anti-Nup214 (red) antibody and analyzed by confocal laser microscopy. Chromatin was stained with DAPI (blue). Scale bar, 10 μm. The white arrow indicates a typical multinucleated cell. (D) Mitotic HeLa cells were scored for multinucleated cell defects. The data represent the means of three experiments in which 250 mitotic cells were scored in three independent experiments.

Since Nup88 is overexpressed in many cancer patients [21,22], we hypothesized that the reason for Nup88-associated tumorigenesis may be related to disruption of Nup88-Nup214 interactions during interphase or mitosis. To test this hypothesis, we altered their balance *in vivo *by modulating their concentrations using RNAi and overexpression strategies, and assayed the effects on cell morphology and spindle polarity. We expressed full-length Nup88 fused to GFP (Figure 2A) in HeLa cells and examined its effects on the progression of mitosis. We found that 23% of transfected cells (counting transfected cells with GFP signals: n = 250 in three independent experiments) were multinucleated among GFP-Nup88-transfected cells compared with only 5% among GFP control vector-transfected cells (Figure 2B and 2D). Nup214 staining was still colocalized with Nup88 staining in multinucleated cells (Figure 2C). Given our observation of Nup88-Nup214 interactions during mitosis, we explored the effects of Nup214 coexpression with Nup88 in rescuing the multipolar spindle phenotype of Nup88-overexpressing cells. Since the Nup214_684-974 _fragment was reported to bind to Nup88 in cells [17], we examined the effects of expressing this fragment in HeLa cells. If Nup88-Nup214 interactions are biologically functional, the Nup214_684-974 _fragment should sequester endogenous Nup88 and rescue the multinucleated phenotypes. Indeed, when Nup88 and Nup214 levels were simultaneously increased by transient overexpression, the incidence of multinucleated cells were greatly reduced compared with cells expressing the control DsRed vector (counting transfected cells with fluorescent signals: n = 250 in three independent experiments) (Figure 2D). Consistent with clinical observations [20-22], we found that overexpressed Nup88 enhanced multinucleated cell formation, leading to aneuploidy, enhanced genomic instability and tumorigenesis in cancer cell lines. Nup214 interacted with Nup88 during mitosis and Nup214_684-974 _partially rescued the Nup88 overexpression defects after co-transfection (Figure 2D).

**Figure 3:**
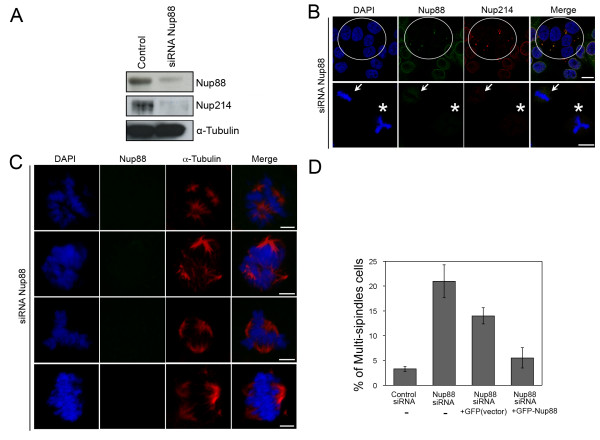
**Depletion of Nup88 causes multipolar spindles**. (A) Effects of Nup88 depletion on protein levels. Lysates of control siRNA-transfected HeLa cells and Nup88 siRNA-transfected HeLa cells at 72 h after transfection were analyzed by immunoblotting with the indicated antibodies. (B) Knockdown of Nup88 by siRNA leads to abnormal chromosome formation in mitosis. HeLa cells were transfected with a siRNA duplex against Nup88. After 72 h, the cells were stained with anti-Nup88 (green) and anti-Nup214 (red) antibodies. Chromatin was visualized using DAPI (blue). Confocal microscopy of Nup88 siRNA-treated cultured cells reveals a loss of nuclear envelope-associated Nup88 (white circle). Fluorescence intensity measurements performed on randomly selected interphase cells (Total) indicate a ≈70% reduction in the Nup88 level (± 5%; P < 0.001) in siRNA-treated cells versus control (Mock) cells. A larger mean reduction in the anti-Nup88 antibody fluorescence intensity of 80% (± 5%; P < 0.001) is observed when the measurements are restricted to cells containing multipolar spindles. Scale bar, 10 μm. The white asterisk indicates typical chromosome defects. (C) Knockdown of Nup88 by siRNA showed deprived spindle morphology and a significant increase in the frequency of multipolar spindles. The HeLa cells were stained with anti-Nup88 (green) and anti-α-tubulin (red) antibodies. Chromatin was visualized using DAPI (blue). Scale bar, 5 μm. (D) Mitotic HeLa cells were scored for multipolar spindles or cytokinesis defects. The data represents the means of three experiments in which 250 mitotic cells were scored at each time point.

The above results prompted us to examine the consequences of Nup88 depletion. Immunoblotting analysis of HeLa cells subjected to Nup88 siRNA treatment (Additional file 1) for 3 days revealed a ≈85% reduction in Nup88 compared with control cells (Figure 3A). Interestingly, we found that in siRNA-mediated Nup88 knockdown HeLa cells, Nup214 protein levels were also decreased by ~90% from three independent experiments. The same immunoblotted membrane was reprobed for α-tubulin to ensure equivalent loading (Figure 3A). Nup88 knockdown had little, if any, effect on the NPC number, as estimated by immunofluorescence experiments using a variety of anti-nucleoporin antibodies (e.g. m414, data not shown) and chromosome morphology (DAPI staining) during interphase (Figure 3B, 72 h, white circle). Interestingly, when Nup88 knockdown was incomplete, some Nup88 and Nup214 were still localized in spindles during mitosis (Figure 3B, lower panel, white arrow). On the other hand, when Nup88 was almost completely knocked down, Nup214 localization was also abolished from spindles and chromosome separation defects (≈21%; n = 300) were often observed, compared with control siRNA- or control GFP vector-transfected cells (Figure 3B, lower panel, white asterisk). Besides, we also quantified the mitotic defects at 72 h after transfection with siRNA duplexes targeting Nup88 and found that a high proportion (≈25%) of cells displayed strikingly altered spindle morphology compared with control siRNA-transfected cells (transfection efficiency, ≈90%) (Figure 3C). Together, we demonstrated that there was little effect on the nuclear morphology in Nup88 siRNA-treated interphase HeLa cells (Figure 3B, upper panel), however, the multipolar spindle effects on mitotic progression were quite dramatic (Figure 3C). Moreover, to determine whether the observed Nup88 depletion phenotypes are different manifestations of the same defects, or whether mitotic roles of Nup88 can be uncoupled, we employed a rescue strategy by over-expressing GFP-Nup88 in Nup88 knockdown cells or GFP vector alone as control. 24 hours after transfection of GFP-Nup88 into Nup88 RNAi knockdown cells, the multipolar spindles phenotypes were partially rescued (Figure 3D). Indeed, a clear point revealed by this rescue strategy is that Nup88 is the "criminal protein" causing multipolar spindles. In light of these observations, it is worth noting that the Nup88 knockdown was likely to be partial and all RNAi experiments, Nup88 is interpreted with respect to this consideration (almost 95% transfection efficiency was monitored with Block-iT (Invitrogen), data not shown). Taken together, these data suggest that Nup88 knockdown by siRNA enhances chromosome instability and prompts multipolar spindle formation. In any case, our results also provide a useful framework to further examine the dynamics of spindle polarity formation in mitosis and elucidate the roles of Nup88-Nup214 imbalance in chromosome segregation defects leading to aneuploidy.

**Figure 4:**
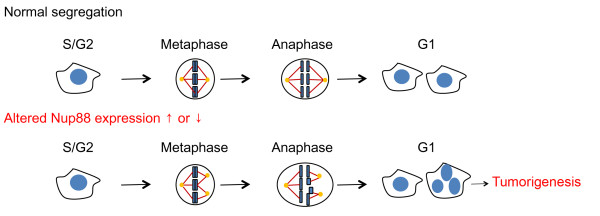
**A model of the relationships of Nup88 with aneuploidy and tumorigenesis**. We propose that proper expression of Nup88 is critical for preventing aneuploidy formation and tumorigenesis.

Gain or loss of whole chromosomes is often observed in cell from cancer patients and is thought to be caused by aberrant chromosome segregation during mitosis. Errors in chromosome segregation are the main source of aneuploidy and a driving force in tumor development. Here, we have clearly demonstrated that alterations in the expression of the tumor marker Nup88 *in vivo *by modulating its concentration using RNAi and overexpression strategies enhanced multinucleated cells and multipolar spindle formation, leading to aneuploidy and enhanced genomic instability. A possible explanation for the appearance of these cell populations is that disruption of the normal Nup88 expression level (by overexpression or depletion strategies) causes a failure in the kinetochore-spindle microtubule interactions to capture chromosomes, eventually leading to mitotic exit and nuclear envelope reformation around dispersed chromosomes or groups of chromosomes. In this way, the defective prometaphase/metaphase cells could represent the precursors of the multinucleated cells (Figure 4). In light of the above data, we propose a speculative model for the tumorigenesis of Nup88 during mitosis (Figure 4).

## Competing interests

The authors declare that they have no competing interests.

## Authors' contributions

RW designed and CH and NH performed research and analyzed data. RW supervised the study and wrote the paper. KY contributed to research reagents and discussions. All the authors have read and approved the final manuscript.

## Supplementary Material

Additional file 1 Materials and Methods. Click here for file
