# Supplementary material for: Characterization of the role of the tumor marker Nup88 in mitosis
Source: Mol Cancer. 2010 May 24;9:119. doi: 10.1186/1476-4598-9-119 (PMC2890605; doi:10.1186/1476-4598-9-119)
Supplement: Additional file 1 — Materials and Methods. [file 1476-4598-9-119-S1.DOC]

**Supplementary Text** (**Materials and Methods**)

**Plasmids**

The plasmid encoding full-length human Nup88 (Open Biosystems, Huntsville, AL) was subcloned into pEGFP-C1 (Clontech, ) with a GFP tag. The Nup214_684-974_ domain was subcloned by PCR from pET28a-Nup214_684-974_ (Blobel Laboratory, Rockefeller University) into the pDsRed-Monomer-C1 vector (Clontech). All cloning procedures were essentially carried out as described previously [2-3]. Sequence analyses were performed at the DNA sequencing facility of the Kanazawa University Cancer Research Institute using a PRISM3100-AvantGenetic Analyzer (Applied Biosystems, Foster City, CA). DNA and protein databases were searched using the BLAST or BLAT search algorithms at the NCBI.

**Immunoprecipitation**

For immunoprecipitation analyses, approximately 1×10^7^ cells were seeded and synchronized as described [2-3]. Mitotic HeLa cells were collected, washed with PBS, centrifuged at 400 × *g* for 10 min and lysed in 1 ml of cold lysis buffer (50 mM Tris-HCl pH 7.2, 250 mM NaCl, 0.1% Nonidet P-40, 2 mM EDTA, 10% glycerol) containing 1× protease inhibitor mixture (Roche) and 1 mM PMSF. The lysates were centrifuged at 14,000 × *g* for 30 min at 4°C. The resulting supernatants were precleared with 50 μl of Protein A/G bead slurry (Santa Cruz Biotechnology, Santa Cruz, CA), mixed with 10 μl of various antibodies, and incubated for 1 h at 4°C with rocking. The beads were then washed five times with 500 μl of lysis buffer. After the last wash, 50 μl of 1× SDS/PAGE blue loading buffer (New England Biolabs,) was added to the beads before loading onto SDS-PAGE gels.

# Cell culture, transfections, and synchronization and siRNA assays

# HeLa cells were transfected with control or Nup88 siRNAs, GFP-Nup88 and pDsRed-Nup214_684-974_ plasmids using Lipofectamine 2000 (Invitrogen), according to the manufacturer’s protocols. Cells were synchronized in S phase by double thymidine block using 2 mM thymidine with the following modifications [1,2]. In experiments involving siRNA oligonucleotides, the cells were transfected at 24 h before the initiation of the first thymidine block, and then collected or imaged after 72 h. For the RNAi experiments, siRNA duplexes targeting Nup88 and control siRNA were purchased from Santa Cruz Biotechnology. Transfection efficiency was monitored with Block-iT (Invitrogen).

# Antibodies and immunofluorescence

The anti-α-tubulin monoclonal antibody DM1A (CT9026) was obtained from Sigma-Aldrich (St. Louis, MO). The anti-Nup88 antibodies were purchased from Santa Cruz Biotechnology (sc-98351) and BD Biosciences (San Jose, CA; 611896). The anti-Nup214 antibody was from Abcam (Cambridge, UK; ab70497) and the anti-CENP-E antibody was from Santa Cruz Biotechnology (sc-56286). Secondary antibodies were obtained from Molecular Probes (Eugene, OR). For immunofluorescence, synchronized HeLa cells were washed in PBS and fixed for 10 min in 4% PFA in PBS. The cells were then permeabilized with 0.2% Triton X-100 in PBS for 10 min at room temperature. Samples were mounted onto coverslips with ProLong Gold Antifade reagent (Invitrogen) and examined using an LSM5 EXCITER confocal microscope (Carl Zeiss, Jena, Germany). All images were acquired using a plan-Apochromat 63× (NA, 1.4) objective or 100× (NA, 1.4) objective.
